# Efficacy of low-protein diet in diabetic nephropathy: a meta-analysis of randomized controlled trials

**DOI:** 10.1186/s12944-019-1007-6

**Published:** 2019-04-01

**Authors:** Xiao-Feng Li, Jing Xu, Ling-Jiao Liu, Fang Wang, Sheng-Lin He, Ya Su, Chun-Ping Dong

**Affiliations:** 1 Department of Endocrinology, Shanxi Provincial People’s Hospital, No. 256 West Youyi Road, Xi’an, 710068 China; 2 Department of Endocrinology, The Second Affiliated Hospital of Xi’an Jiaotong University, Xi’an, 710004 China; 3 Department of Endocrinology, Xi’an Central Hospital, Xi’an, 710004 China

**Keywords:** Diabetic nephropathy, Low protein diet, RCT, Meta-analysis

## Abstract

**Purpose:**

We aimed to systematically assess the efficacy of low-protein diet preventing progression of diabetic nephropathy based on randomized controlled trials (RCTs).

**Methods:**

A systematic and electronic search was conducted. Initial searches of literature updated to September 2018 were made using the following databases including CNKI, VIP, Wanfang, Cochrane, PubMed, and Embase using the index words for qualified RCTs. Additional searches were performed to identify linked literature sources. Data of RCTs on low-protein diet versus control diet, efficacy analysis of kidney function, nutritional status or proteinuria were extracted. Random effects model and fixed effects model were applied to combine the data which were further analyzed by Chi-squared test and I^2^tests. The main outcomes were then analyzed through the use of relative risks (RR), mean difference (MD) and its 95% confidence interval (95% CI).

**Results:**

Twenty articles were included in the present meta-analysis with a total of 690 patients in the low-protein diet group (LPD) and a total of 682 patients in the control group. Moderate to strong evidence indicated that LPD was significantly effective for decreasing the urinary albumin excretion rate (SMD:0.62, 95%CI:0.06–1.19) and proteinuria (SMD:0.69, 95%CI:0.22–1.16) versus the control group. No statistical difference, however, was found in glycosylated hemoglobin (SMD:0.17, 95%CI:-0.18–0.51), serum creatinine (SMD:0.20, 95%CI:-0.26–0.66), as well as glomerular filtration rate (SMD:0.21, 95%CI:-0.29–0.71) between the two groups.

**Conclusion:**

The current meta-analysis reveals an effective role of low-protein diet in improving diabetic nephropathy. However, the small number of involved patients may limit the accuracy of results. High-quality RCTs with a larger sample size in the future are required to confirm the current findings.

## Introduction

Diabetes is a highly prevalent chronic disease constitutes a major public health issue and inflicts a severe financial burden on the society and family. About 40% of diabetes patients would develop diabetic nephropathy [1]. Diabetic nephropathy is associated with a high risk of mortality with cardiovascular disease as a strong independent risk factor [2, 3]. Besides, diabetic nephropathy associated with type 1 as well as type 2 diabetes mellitus is considered a leading cause of end-stage renal disease worldwide [4, 5]. Blood pressure control and optimal glycaemic control can slow down the progression of diabetic nephropathy through renin-angiotensin system blockade. Therefore, it is important to search for approaches to decelerate diabetic nephropathy progression.

According to Diabetes and Nutrition Study Group of the European Association, the Study of Diabetes suggests that the dietary approach for weight loss and treating diabetes is a low-fat, high-carbohydrate, and energy-deficient diet [6, 7]. Earlier animal experiment and humans studies supported by American Diabetes Association recommendslow-protein diet (LPD) as a dietary approach in clinical guidelines [8]. LPD is proven to have beneficial effect in decreasing the progression of renal disease as well as improving survival rate in patients harboring various glomerulopathies, such as diabetic kidney disease [9, 10]. However, controversy exists as several studies showed no significant benefit of LPD in slowing down the progression of diabetic nephropathy.

The present meta-analysis aims to summarize current available evidence based on RCTs, and to determine the efficacy profile of LPD in terms of diabetic nephropathy progression.

## Methods

### Literature search

An electronic literature search was conducted for eligible RCTs through the use of Weipu (VIP), WangFang, CNKI (China National Knowledge Infrastructure), PubMed, the Cochrane library, and Embase updated to Sep 2018. In addition, we searched related publications as well as reference materials. The search process was carried out separately by two reviewers. Any differences were settled through the aid of a third party. Ethics approval was waived for this study because the study involved no human participants or animals.

### Selection criteria

To be included in the current meta-analysis, studies should meet the following criteria: [1] RCTs; [2] patients had type 1 or 2 diabetic nephropathy; [3] patients received LPD or normal protein diet; [4] at least one clinical outcome was reported for analysis; [5] publications were English or Chinese.

Studies that met the following criteria should be excluded: [1] duplicate publication, or shared result or content; [2] incomplete or incorrect data; [3] case report, expert comment, systematic review, conference report, meta analysis, theoretical research, and economic analysis; [3] irrelevant or no outcomes.

All the present studies were manually screened separately by two reviewers for evaluation of eligibility. Any arising disagreements were then settled through the help of a third reviewer.

### Data extraction

The authors extracted data from the included studies. The present study consisted of basic information and main outcomes. Basic information contained the following parameters: the author’s name, sample size, interventions of the treatment and control group, percentage of male subjcts, body mass index (BMI), mean age, duration of diabetes, and type of diabetes. The second part contained clinical outcomes: glycosylated hemoglobin, urinary albumin excretion rate, serum creatinine, glomerular filtration rate, and proteinuria. We appraised the quality of current trials and studies with the use of the Jadad scoring checklist and all the RCTs were evaluated based on the following five items: appropriateness of generating randomized sequence, statement of randomization, use of double blinding, detail of withdrawals and dropouts and description of double blinding method. A score less than 3 in the included studies represented a low-quality and high bias risks, and a score greater than 3 represent a trial with high quality. The above mentioned process was separately conducted by two investigators; arising differences were resolved by discussion to reach a consensus.

### Statistics analysis

The meta-analysis was conducted through the use of the STATA 10.0 (TX, USA). Heterogeneity of the trial results was assessed with the Chi-squared and I^2^ tests to select ideal analysis model (random-effects model or fixed-effects model): I^2^ > 50% and Chi-squared test *P* ≤ 0.05 reflected high heterogeneity and the random-effects model was utilized; I^2^ ≤ 50% and Chi-squared test *P* > 0.05 reflected acceptable heterogeneity data and the fixed-effects model was used. As for continuous variables, they were initially expressed as mean ± standard deviation and then analyzed through the use of mean differences (MD). Categorical data was expressed in percentages and further analyzed through odds ratio (OR) or relative risk (RR). MD with its 95% CI was used to analyze glycosylated hemoglobin, urinary albumin excretion rate, serum creatinine, glomerular filtration rate, and proteinuria. To identify the publication bias, we utilized the funnel plot, Begg and Egger’s weighted test.

## Results

### Study characteristics

**Fig. 1:**
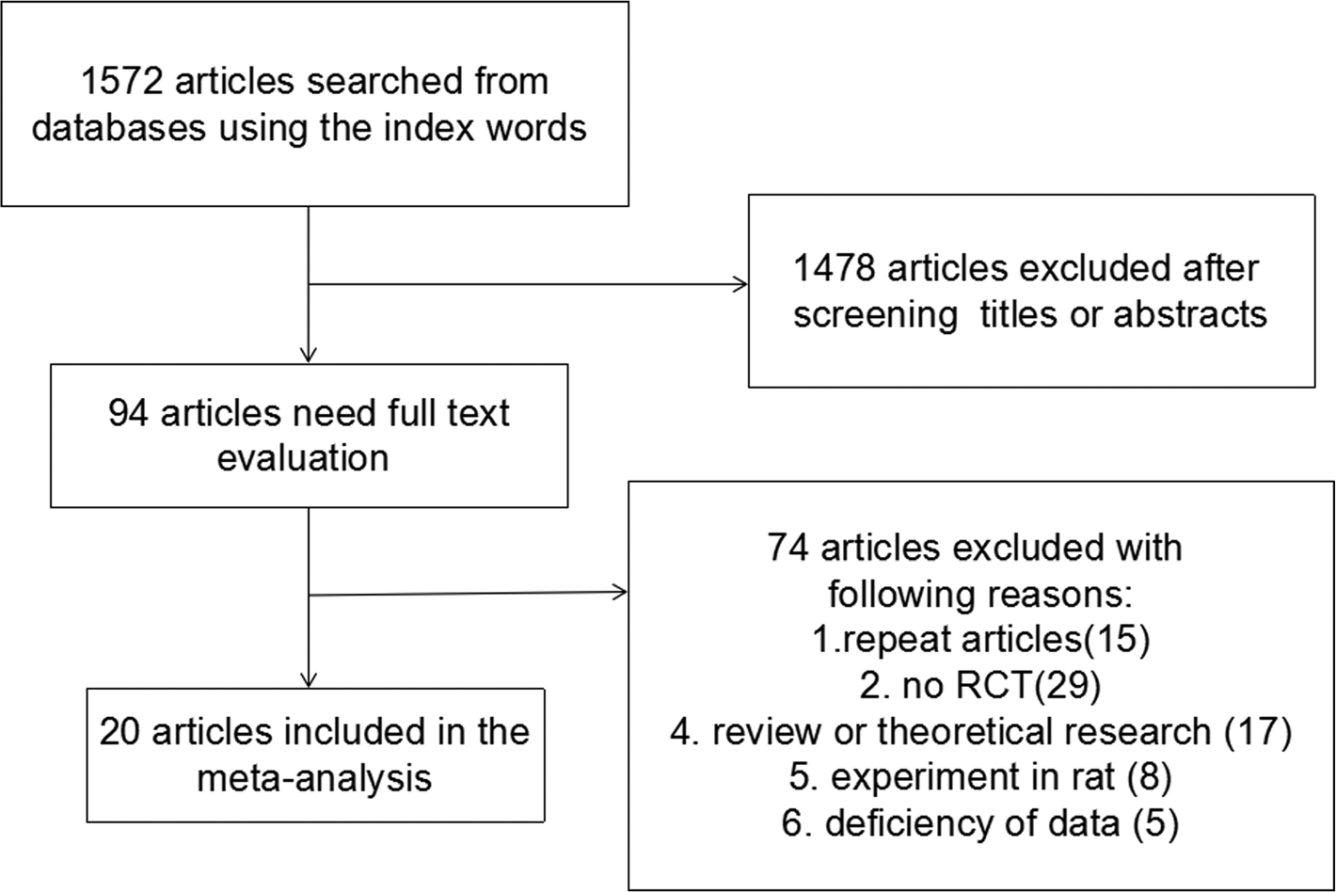
The flow diagram of the literature search process

Through search using the index words, a total of 1572 publications were included. After title and abstract screening, 1478 publications were then excluded; thus, 94 publications were left for further assessment. During full-text screening, 74 publications were excluded due to duplicated publications [15], non-RCTs [29], review or theoretical research [17], animal studies [8], or insufficient data [5]. Therefore, a final total of 20 studies [11–30] were included in the current meta-analysis, of which 690 and 682 patients were studied and evaluated in the LPD group and control group respectively (Fig. 1).

Table 1 lists the major characteristics of studies. The basic information consisted of sample size, male, age, BMI, duration of diabetes, type of diabetes, inventions of the LPD group and the control group, and Jadad score. Nine studies analyzed type 2 diabetes patients, two studies for type 1 diabetes, and two studies for both type 1 and 2 diabetes; seven studies did not specifiy type of diabetes. In the LPD group, 16 studies reported that the protein intake was between 0.6 g/kg/24 h and 0.8 g/kg/24 h, 3 studies reported that the protein intake was above 0.8 g/kg/24 h, and one study did not provide protein intake data. In the control group, 12 studies reported that the protein intake was above 1.0 g/kg/24 h, and 7 studies just recorded normal or free protein intake. Protein intake was not clearly described in one study. Besides, Table 1 shows the baseline characteristics of the involved population. The mean age ranged from 33 to 72 years, the mean BMI range from 23.3 to 33.6 cm/kg^2^, and the mean duration of diabetes ranged from 7.8 to 28, so the population of the included studies was heterogeneous. The main Jadad score of all the included studies was 2.85. In 13 studies the Jadad score was equal or above 3, and in 7 studies the Jadad score was 2.

**Table 1:** The basic characteristics description of included studies

Study	No. of patients (n)		Male (n)		Age (years)		BMI (kg/m^2^)		Duration of diabetes (years)		Type of diabetes		Jadad score	Inventions	
	LPD	Control	LPD	Control	LPD	Control	LPD	Control	LPD	Control	LPD	Control		LPD	Control
Zhu Ning 2001 a	8	4	-^a^	–	70.0	70.0	23.4	25.1	12.0	12.0	II	II	2	protein intake 0 .6g/kg/24 h, oral hypoglycemic drugs	normal protein intake, oral hypoglycemic drugs
Zhu Ning 2001 b	8	4	–	–	71.0	70.0	24.1	25.1	14.0	12.0	II	II	2	protein intake 0 .8g/kg/24 h, oral hypoglycemic drugs	normal protein intake, oral hypoglycemic drugs
Li Yanping 2000	14	15	8	8	57.0	57.0	–	–	8.2	9.1	–	–	2	protein intake 0 .8g/kg/24 h	protein intake 1.2–1 .5g/kg/24 h
Chen Zhenqian 2013	36	36	18	19	60.6	60.1	24.8	25.0	7.8	8.1	–	–	3	protein intake 0 .8g/kg/24 h	normal protein intake
Yin Qunfang 2009	30	20	18	12	53.6	53.4	–	–	–	–	–	–	2	protein intake 0.6–0 .8g/kg/24 h	protein intake 1 g/kg/24 h
Cui Jirong 2015	100	100	52	45	56.4	58.3	–	–	–	–	II	II	2	protein intake 0.58–0. 82 g/kg/24 h, oral hypoglycemic drugs, inject insulin	normal protein intake, oral hypoglycemic drugs, inject insulin
Tao Jianxun 2008	66	68	44		60.5	58.0	24.3	24.5	9.1	10.9	II	II	2	protein intake 0 .7g/kg/24 h	protein intake 1 g/kg/24 h
H.Makino.K.Shikata 2009	56	56	33	33	57.5	56.3	–	–	–	–	II	II	3	protein intake 0.92 ± 0. 43 g/kg/24 h	protein intake 1.22 ± 0. 25 g/kg/24 h
Bertrand Dussol 2005	22	25	19	20	52.0	63.0	28.0	27.0	15.0	20.0	I、II	I、II	3	protein intake 0 .8g/kg/24 h	protein intake 1 .2g/kg/24 h
Carlo Meloni 2004	40	40	22	19	52.7	56.3	–	–	–	–	I、II	I、II	3	protein intake 0 .8g/kg/24 h	free protein intake
G.D.Brinkworth 2004	19	19	7	8	62.7	60.9	33.3	33.6	–	–	II	II	3	–	–
Henrik P.Hansen 2002	41	41	30	23	40.0	41.0	25.0	25.0	27.0	28.0	I	I	3	protein intake 0. 89 g/kg/24 h	protein intake 1. 02 g/kg/24 h
LTJ Pijls 2002	63	68	40	36	63.0	65.0	27.4	28.2	6.7	7.2	II	II	4	protein intake 0 .8g/kg/24 h	free protein intake
Loek T.J.Pijls 1999	58	63	39	35	64.0	63.0	27.3	28.1	6.8	7.2	II	II	4	protein intake 0 .8g/kg/24 h	free protein intake
Frederick J Raal 1994	11	11	3	5	29.0	30.0	26.0	23.8	20.0	21.0	–	–	3	protein intake 0 .8g/kg/24 h	Protein intake > 1 .6g/kg/24 h
Robin P.F.Dullaart 1993	14	16	14	13	43.0	39.0	25.1	23.3	23.0	20.0	–	–	4	protein intake 0 .6g/kg/24 h	free protein intake
Kathleen Zeller 1991	20	15	11	10	33.0	35.0	–	–	21.0	22.4	I	I	3	protein intake 0 .6g/kg/24 h	protein intake 1 g/kg/24 h
Adolfo Ciavarella 1987	7	9	4	5	37.6	36.8	–	–	16.6	18.7	–	–	2	protein intake0.71 ± 0. 12 g/kg/24 h	protein intake 1.44 ± 0. 12 g/kg/24 h
Benh.Brouhard 1990	8	7	–	–	36.0	30.0	–	–	19.0	19.0	–	–	2	protein intake 0 .6g/kg/24 h	protein intake 1 g/kg/24 h
L.Velazquez Lopez 2008 a	9	10	7	17	68.0	66.3	–	–	18.7	15.0	II	II	4	protein intake 0.6–0 .8g/kg/24 h	protein intake 1–1 .2g/kg/24 h
L.Velazquez Lopez 2008 b	10	12			68.0	66.3	–	–	18.7	15.0	II	II	4	protein intake 0.6–0 .8g/kg/24 h	protein intake 1–1 .2g/kg/24 h
L.Velazquez Lopez 2008 c	10	9			68.0	66.3	–	–	18.7	15.0	II	II	4	protein intake 0.6–0 .8g/kg/24 h	protein intake 1–1 .2g/kg/24 h
Mauro Giordano 2014	40	34	–	–	72.0	71.0	30.8	31.4	–	–	II	II	3	protein intake 0 .7g/kg/24 h	protein intake 1 .1g/kg/24 h

^a^: the studies has not provided this data; *BMI* body mass idex, *LPD* low-protein diet, *I* type 1 diabetes; *II* type 2 diabetes

### Quality assessment and potential bias

**Fig. 2:**
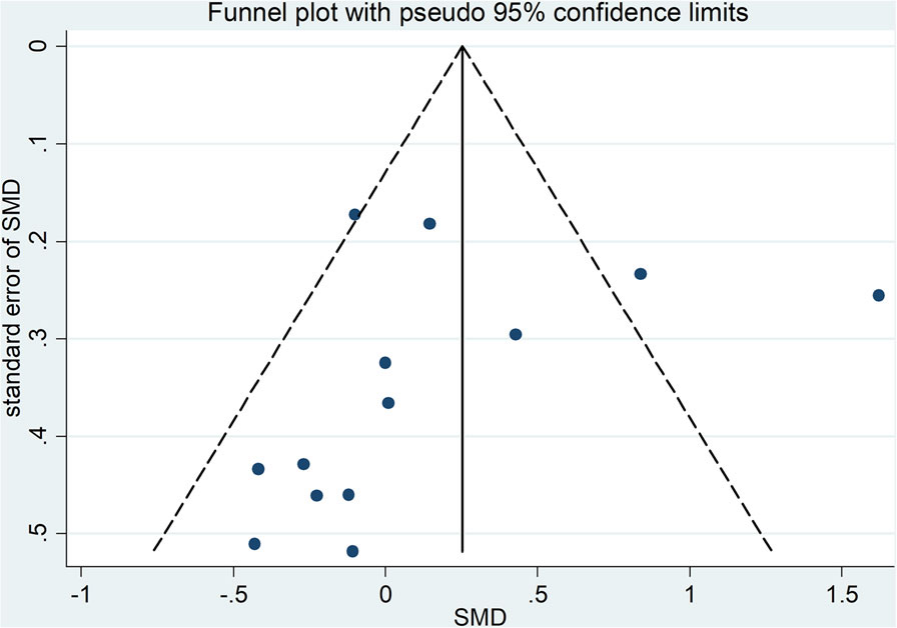
Funnel plot of studies included in the meta-analysis

We applied funnel plot, Egger’s test, and Begg and Mazumdar’s rank test, and for the quality assessment as well as for potential bias. Notable dissymmetry was found according to the funnel plot for SMD in glycosylated hemoglobin, indicating significant publication bias (Fig. 2). In addition, we found significant asymmetry with the application of Begg and Mazumdar’s rank test (Z = 1.28, *p* = 0.200). There was a significant publication bias on basis of the Egger’s test result (*p* = 0.415).

### Effect of LPD on glycosylated hemoglobin

**Fig. 3:**
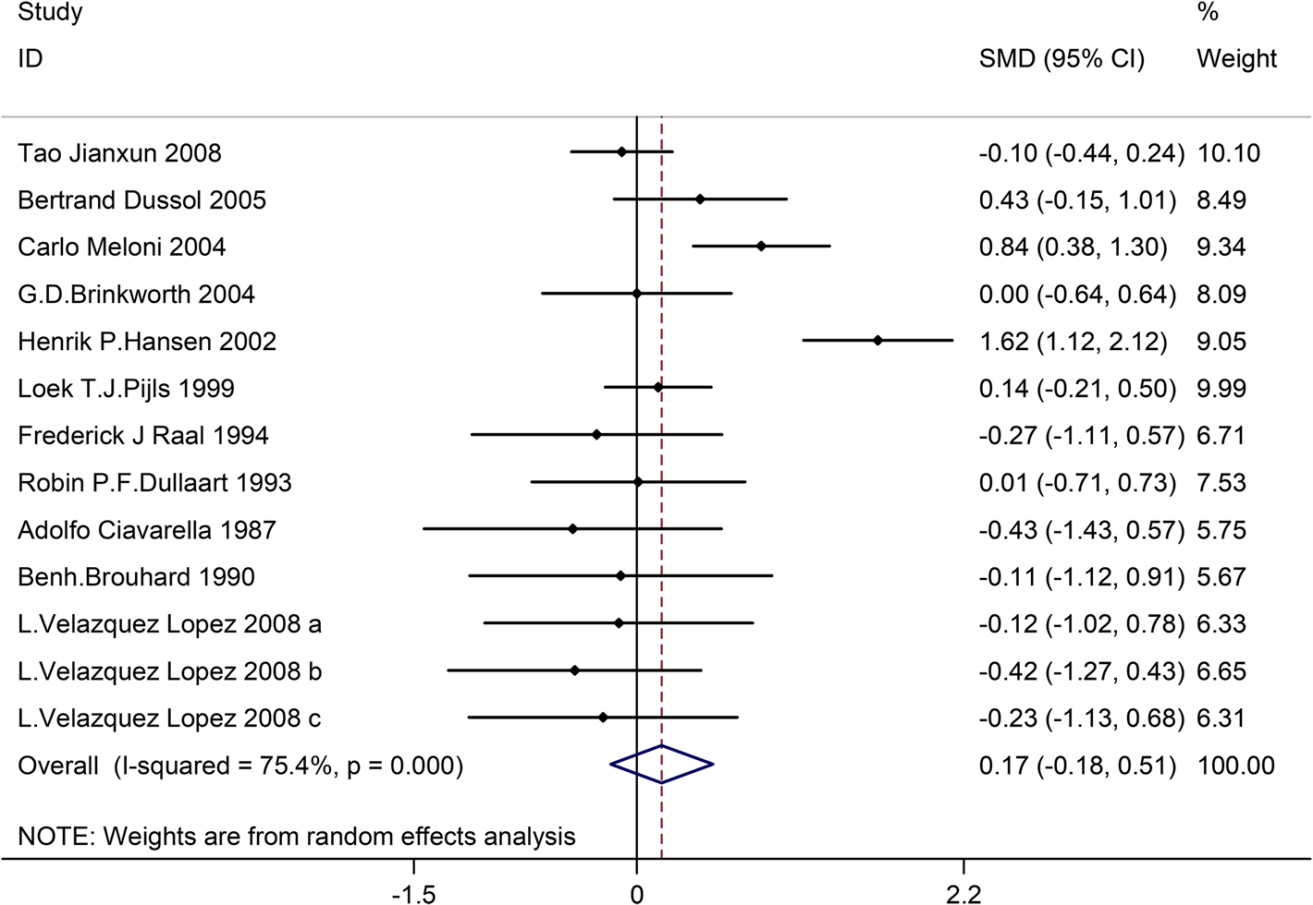
Effects of low-protein diet on glycosylated hemoglobin

Thirteen trials involving 645 patients (the LPD group = 315, the control group = 330) reported the effect of LPD on glycosylated hemoglobin. According to the I^2^tests-value (I^2^ = 75.4%) and Chi-squared test *P*-value (*P* = 0.000), the random effects model was applied to analyze glycosylated hemoglobin. No significant difference in glycosylated hemoglobin was found in the pooled results between the LPD and the control group (SMD:0.17, 95%CI:-0.18–0.51) (Fig. 3).

### Effects of LPD on urinary albumin excretion rate

**Fig. 4:**
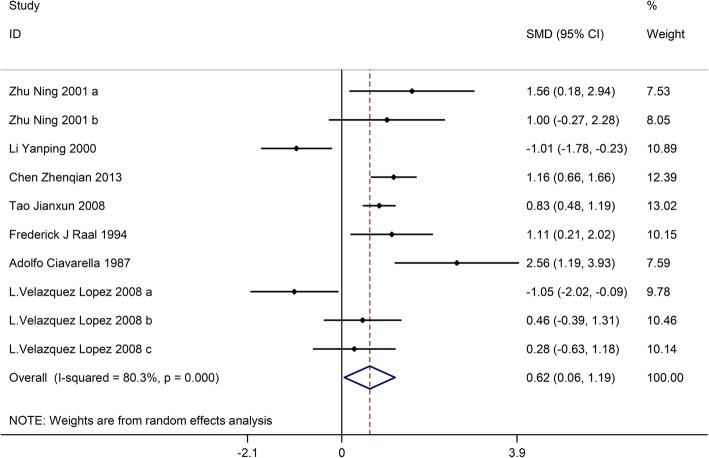
Effects of low-protein diet on urinary albumin excretion rate

Ten trials with a total of 357 patients (the LPD group = 179, and the control group = 178) showed the effect of LPD on urinary albumin excretion rate. On the basis of I^2^tests-value (I^2^ = 80.3%) and Chi-squared test *P*-value (*P* = 0.000), the random effects model was applied to analyze urinary albumin excretion rate. The pooled results showed the urinary albumin excretion rate was significantly decreased in the LPD group versus the control group (SMD:0.62, 95%CI:0.06–1.19) (Fig. 4).

### Effects of LPD on serum creatinine

**Fig. 5:**
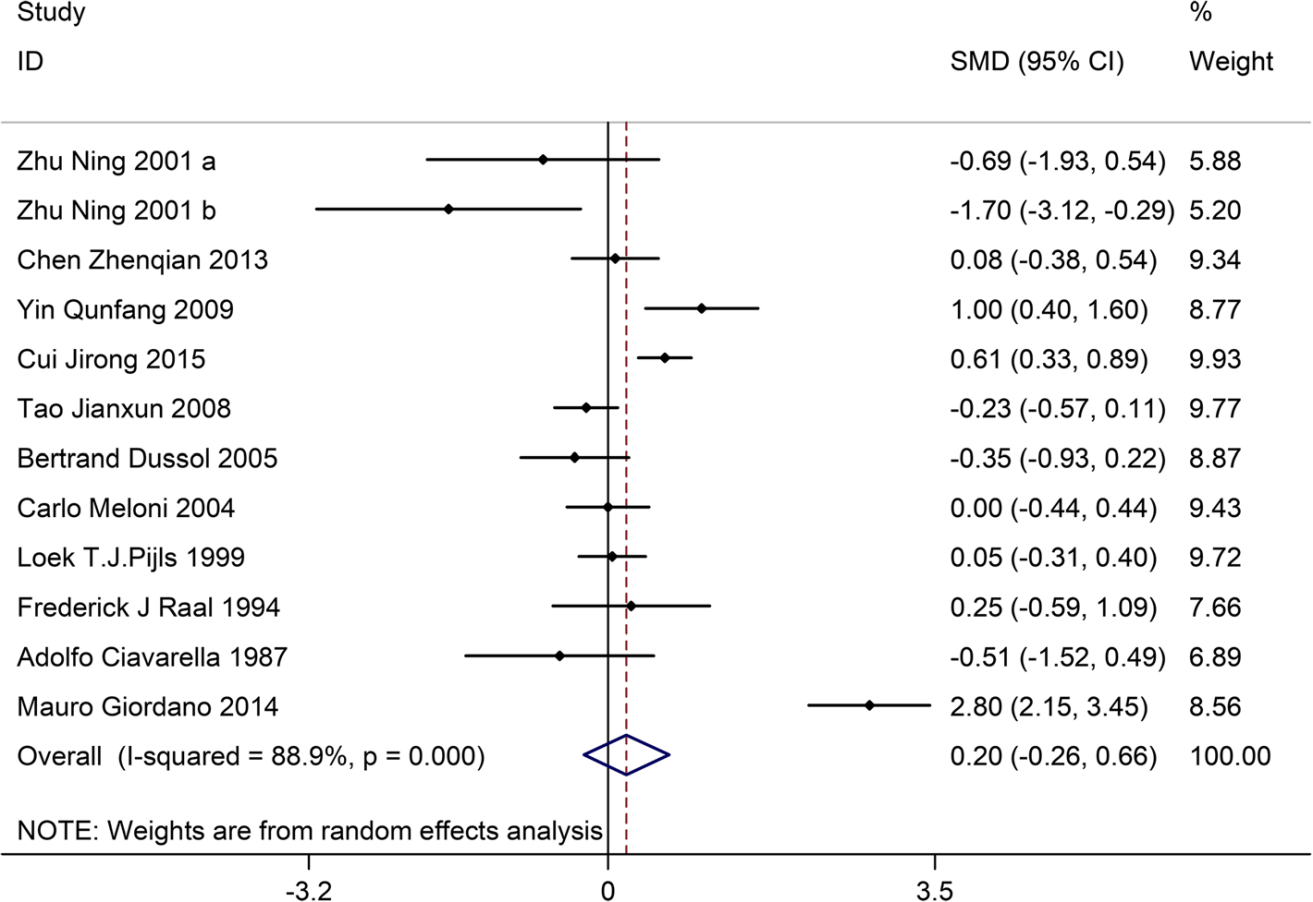
Effects of low-protein diet on serum creatinine

Twelve trials involving 840 patients (the LPD group = 426, and the control group = 414) showed the effect of LPD on serum creatinine. On the basis of the I^2^ test-value (I^2^ = 88.9%) and Chi-squared test *P*-value (*P* = 0.000), the random effects model was utilized to analyze serum creatinine. No significant difference was found in the pooled results of serum creatinine between the LPD group and the control group (SMD:0.20, 95%CI:-0.26–0.66) (Fig. 5).

### Effects of LPD on glomerular filtration rate

**Fig. 6:**
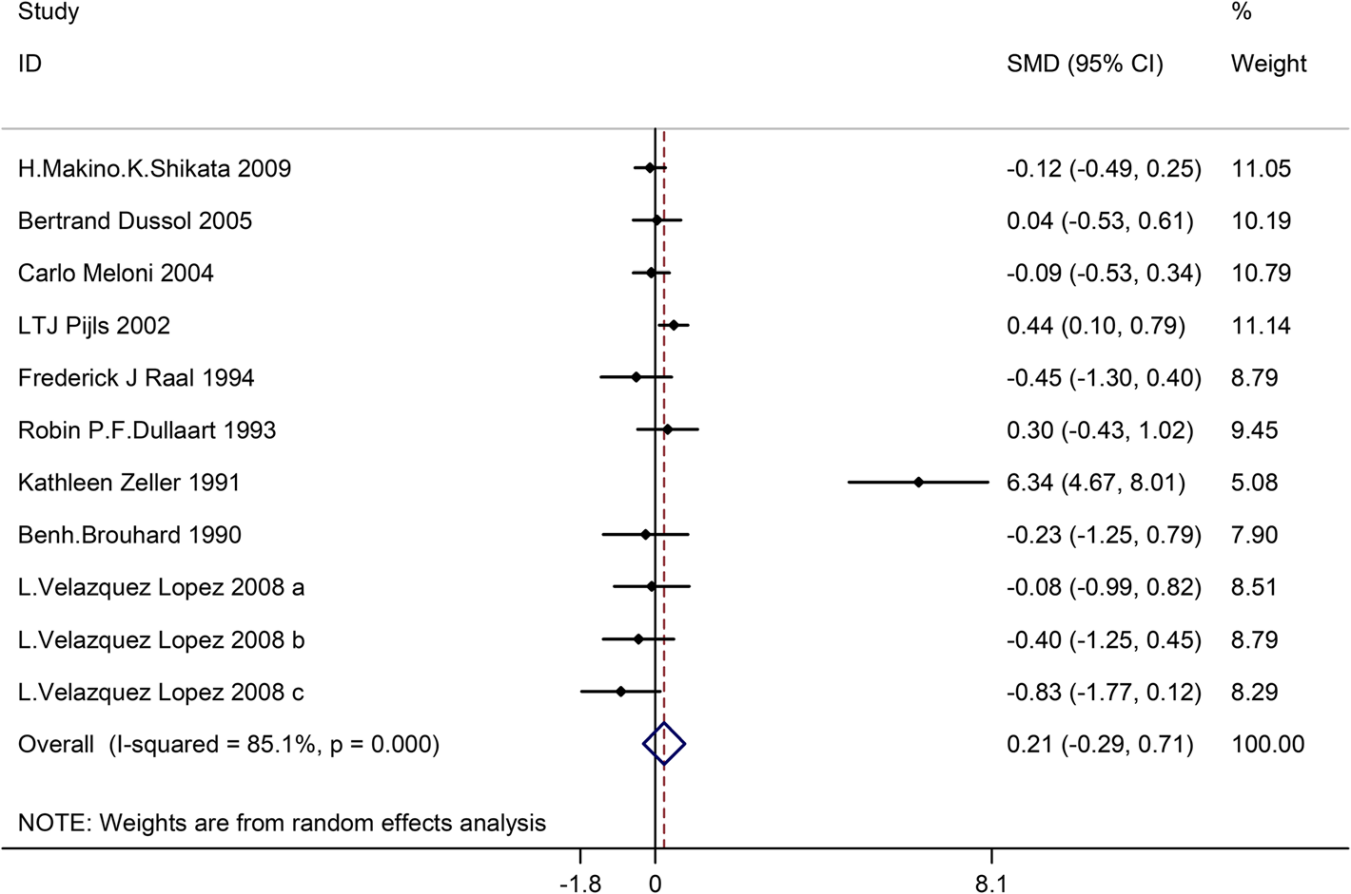
Effects of low-protein diet on glomerular filtration rate

Twelve trials involving732 patients (the LPD group = 363, and the control group = 369) showed the effect of LPD on glomerular filtration rate. On the basis of I^2^test-value (I^2^ = 85.1%) and Chi-squared test *P*-value (*P* = 0.000), we analyzed glomerular filtration rate through the random effects model. No significant difference was identified among the pooled results of glomerular filtration rate between the LDP group and the control group (SMD:0.21, 95%CI:-0.29–0.71) (Fig. 6).

### Effects of LPD on proteinuria

**Fig. 7:**
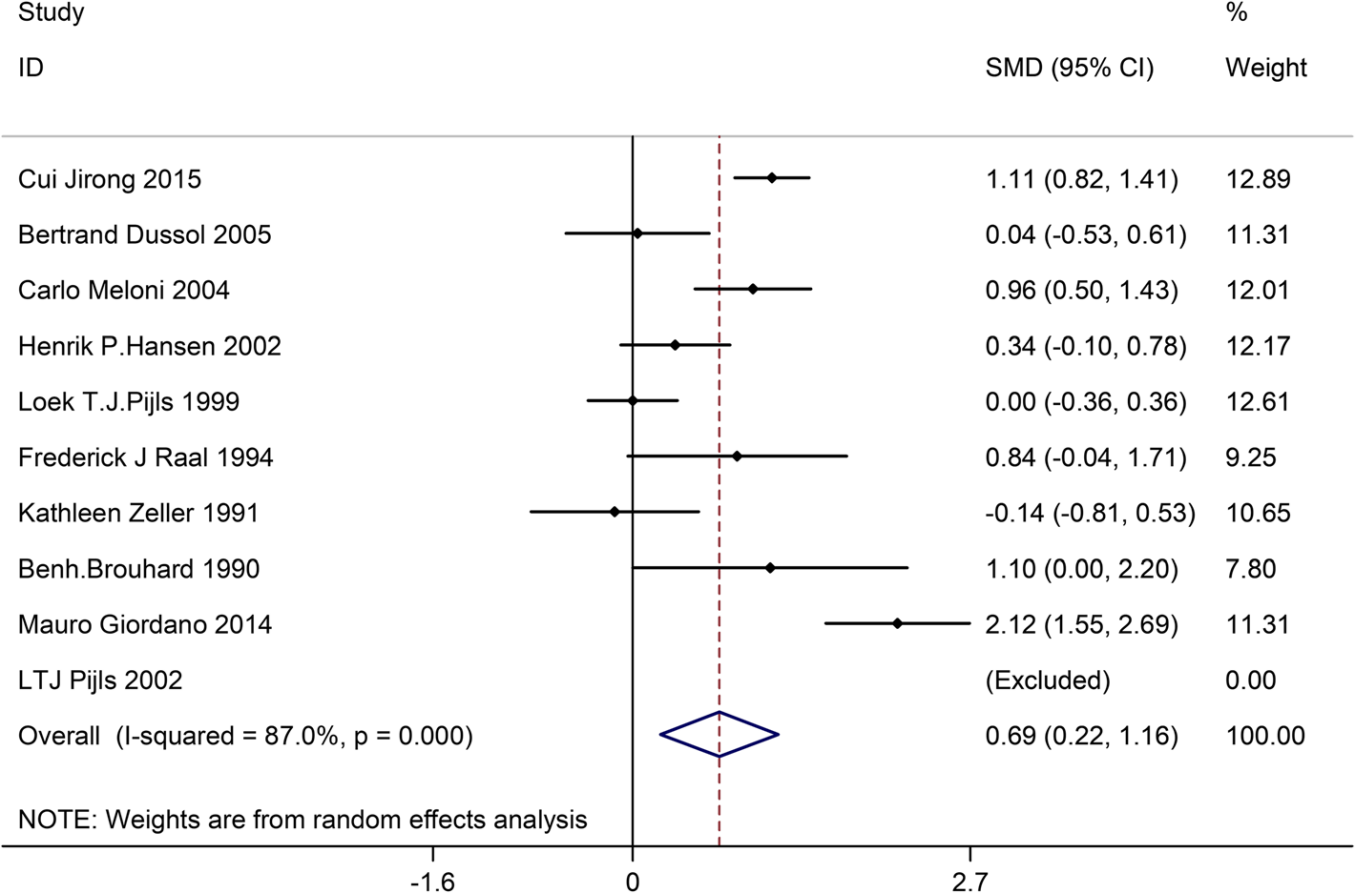
Effects of low-protein diet on proteinuria

Ten trials with a total of 807 patients (the LPD group = 403, and the control group = 404) showed the effect of LPD on proteinuria. According to I^2^ test-value (I^2^ = 87.0%) and Chi-squared test *P*-value (*P* = 0.000), we analyzed proteinuria using the random effects model. The pooled results showed that proteinuria was obviously decreased in the LPD group versus the control group (SMD:0.69, 95%CI:0.22–1.16) (Fig. 7).

## Discussion

As a matter of fact, a total of three meta-analyses on the current topic were published with pooled data from RCTs. One meta-analysis by Pedrini et al. [16] showed beneficial effects of LPD. Nevertheless, they combined non-randomized crossover trials with RCTs. Furthermore, aggregated outcomes of albuminuria or GFR have been utilized. According to the meta-analyses by Pan et al. and Robertson et al., there was no remarkable efficacy in terms of kidney function. The different results may be due to difference in population size and the number of pooled studies. In addition, earlier meta-analysis by Robertson et al. pooled data from only seven RCTs, focusing on T1DM patients in their study. The studies are in consistent with earlier meta-analysis given that there was no statistical significance with improved GFR in T1DM patients. Pan et al. conducted a meat analysis that included two reports by Pijls et al. on patients with identical baseline characteristics (Table 1). The intervention period and number of patients were different, which was longer and larger in a recent publication. After discussion, the reviewers believe and considered the previous publicationsas the interim analysis of a longer project. Hence, the results were not used simultaneously for analysis of the same outcome despite that both studies were listed in our meta-analysis. The data on albuminuria and GFR were extracted from recent publications, and HbA1c from previous publications due to lack of recent studies.

The current meta-analysis extends efforts with an attempt to confirm the efficacy of LPD in diabetic nephropathy. We conclude the the following advantages of the current study: There showed highly similar baseline characteristics of the LPD group and the control group, and the results proved to be robust according to multiple additional analyses. Additionally, the data were considered to be complete. The drug dispensing process as well as outcomes were recorded accurately; patient loss to follow-up was minimal due to short duration of the studies and a low emigration rate (< 1% per year).

Scicchitano et al. [31] provide an overview of the mechanism of action of nutraceuticals and functional food ingredients on lipids and their role in the management of lipid disorders. Nutraceuticals play a peculiar role in ameliorating human dyslipidemia, but the exact pathophysiological mechanism is still unknown. Functional food ingredients can act on several biochemical pathways able to influence lipid disorders in the human body. Physicians have attempted to identify the mechanisms responsible for nutraceutical actions. From the previous studies, we know that resveratrol, water-insoluble fish proteins, grape seed, curcumin, other nutraceutical and functional food ingredients can play a role in controlling lipid metabolism. In the same way, low-protein diet could limit the protein intake and reduce the metabolic burden.

Admittedly, several limitations of the present analysis should be acknowledged. [1] Only RCTs were included; [2] the predefined criteria were different for patients among various studies; [3] different patients harboring earlier treatments and diseases were unavailable; [4] we included several trials with low quality in the current analysis; [5] protein intake was different in the inventions of the LPD group and the control group were among different studies (the detailed information is presented in Table 1); [6] age, BMI, duration of diabetes, and type of diabetes were different among various studies, contributing to publication bias; [7] the baseline characteristics (age, BMI, duration of diabetes, percentage of male paitents and type of diabetes) of the study populations were heterogeneous, which could influence the clinical results; [8] the number of involved patients was small; [9] we used the pooled data for analysis;data of individual patients were unavailable, which limits more comprehensive analyses.

Our present meta-analysis provides evidence for modest efficacy of LPD as a diet intervention with significant outcomes on the course of kidney prognosis for patients with diabetic nephropathy. Improved efficacy could be gained with the sustainable intervention and better compliance of patients. Given the results of our study, questions exist considering whether LPD delays or even prevents other more crucial clinical outcomes such as initiation of dialysis, kidney failure, and death. More meta-analyses are warranted in order to focus on the above mentioned outcomes. However, due to the limitations of this study, high-quality studies, large-sample and long-terms should conducted to confirm the conclusions.

## Abbreviations

LPD: low-protein diet
MD: mean difference
OR: odds ratio
RR: relative risk

## References

[1] Ritz E, Orth SR (1999). Nephropathy in patients with type 2 diabetes mellitus. N Engl J Med.

[2] Marks JB, Raskin P (2000). Cardiovascular risk in diabetes: a brief review. J Diabetes Complicat.

[3] Sasso FC, De Nicola L, Carbonara O, Nasti R, Minutolo R, Salvatore T (2006). Cardiovascular risk factors and disease management in type 2 diabetic patients with diabetic nephropathy. Diabetes Care.

[4] Diabetes C, Complications Trial Research G, Nathan DM, Genuth S, Lachin J, Cleary P (1993). The effect of intensive treatment of diabetes on the development and progression of long-term complications in insulin-dependent diabetes mellitus. N Engl J Med.

[5] Retnakaran R, Cull CA, Thorne KI, Adler AI, Holman RR, Group US (2006). Risk factors for renal dysfunction in type 2 diabetes: U.K. prospective Diabetes study 74. Diabetes..

[6] Molitch ME, DeFronzo RA, Franz MJ, Keane WF, Mogensen CE, Parving HH (2003). Diabetic nephropathy. Diabetes Care.

[7] Recommendations for the nutritional management of patients with diabetes mellitus. Eur J Clin Nutr. 2000;54(4):353–5. 10.1038/sj.ejcn.1600962 10747363

[8] American Diabetes A (2013). Standards of medical care in diabetes--2013. Diabetes Care.

[9] Mandayam S, Mitch WE (2006). Dietary protein restriction benefits patients with chronic kidney disease. Nephrology (Carlton).

[10] Kasiske BL, Lakatua JD, Ma JZ, Louis TA (1998). A meta-analysis of the effects of dietary protein restriction on the rate of decline in renal function. Am J Kidney Dis.

[11] Koya D, Haneda M, Inomata S, Suzuki Y, Suzuki D, Makino H (2009). Long-term effect of modification of dietary protein intake on the progression of diabetic nephropathy: a randomised controlled trial. Diabetologia..

[12] Dussol B, Iovanna C, Raccah D, Darmon P, Morange S, Vague P (2005). A randomized trial of low-protein diet in type 1 and in type 2 diabetes mellitus patients with incipient and overt nephropathy. J Ren Nutr.

[13] Meloni C, Tatangelo P, Cipriani S, Rossi V, Suraci C, Tozzo C (2004). Adequate protein dietary restriction in diabetic and nondiabetic patients with chronic renal failure. J Ren Nutr.

[14] Brinkworth GD, Noakes M, Parker B, Foster P, Clifton PM (2004). Long-term effects of advice to consume a high-protein, low-fat diet, rather than a conventional weight-loss diet, in obese adults with type 2 diabetes: one-year follow-up of a randomised trial. Diabetologia..

[15] Hansen HP, Tauber-Lassen E, Jensen BR, Parving HH (2002). Effect of dietary protein restriction on prognosis in patients with diabetic nephropathy. Kidney Int.

[16] Pijls LT, de Vries H, van Eijk JT, Donker AJ (2002). Protein restriction, glomerular filtration rate and albuminuria in patients with type 2 diabetes mellitus: a randomized trial. Eur J Clin Nutr.

[17] Pijls LT, de Vries H, Donker AJ, van Eijk JT (1999). The effect of protein restriction on albuminuria in patients with type 2 diabetes mellitus: a randomized trial. Nephrol Dial Transplant.

[18] Raal FJ, Kalk WJ, Lawson M, Esser JD, Buys R, Fourie L (1994). Effect of moderate dietary protein restriction on the progression of overt diabetic nephropathy: a 6-mo prospective study. Am J Clin Nutr.

[19] Dullaart RP, Beusekamp BJ, Meijer S, van Doormaal JJ, Sluiter WJ (1993). Long-term effects of protein-restricted diet on albuminuria and renal function in IDDM patients without clinical nephropathy and hypertension. Diabetes Care.

[20] Zeller K, Whittaker E, Sullivan L, Raskin P, Jacobson HR (1991). Effect of restricting dietary protein on the progression of renal failure in patients with insulin-dependent diabetes mellitus. N Engl J Med.

[21] Ciavarella A, Di Mizio G, Stefoni S, Borgnino LC, Vannini P (1987). Reduced albuminuria after dietary protein restriction in insulin-dependent diabetic patients with clinical nephropathy. Diabetes Care.

[22] Brouhard BH, LaGrone L (1990). Effect of dietary protein restriction on functional renal reserve in diabetic nephropathy. Am J Med.

[23] Velazquez Lopez L, Sil Acosta MJ, Goycochea Robles MV, Torres Tamayo M, Castaneda Limones R (2008). Effect of protein restriction diet on renal function and metabolic control in patients with type 2 diabetes: a randomized clinical trial. Nutr Hosp.

[24] Giordano M, Ciarambino T, Castellino P, Cataliotti A, Malatino L, Ferrara N (2014). Long-term effects of moderate protein diet on renal function and low-grade inflammation in older adults with type 2 diabetes and chronic kidney disease. Nutrition..

[25] Zhu N (2001). Low-protein diet and kidney function on non-insulin-dependent diabetic nephropathy. Med J Chin PLA.

[26] Chen ZQ (2013). Effect of low-protein diet on renal function of early diabetic nephropathy. Journal of Hygiene Research.

[27] Li YP, Li YC (2000). Effect of low-protein diet on renal function of diabetic nephropathy. Med J Harbin.

[28] Yi QF (2009). The method of low-protein diet of diabetic nephropath in clinical stage. Guangdong Medical Journal.

[29] Cui RJ (2015). Explore the efficacy of low-salt and low-protein in treatment of diabetic nephropathy. Contemporary Medicine Forum.

[30] Tao JX (2008). Affection of low protein on the patients with early diabetic nephropathy type 2. Journal of Surgeon in Southwest China.

[31] Scicchitano P, Cameli M, Maiello M, Modesti PA, Muiesan ML, Novo S (2014). Nutraceuticals and dyslipidaemia: beyond the common therapeutics. J Funct Foods.

